# Responsive Feeding Practices to Promote Healthy Diets: A Mixed Method Study among Low-Income Caregivers with Toddlers

**DOI:** 10.3390/nu16060863

**Published:** 2024-03-16

**Authors:** Kate E. Killion, Amy Corcoran, Maria J. Romo-Palafox, Jennifer L. Harris, Inna Kagan, Laura Gilbert, Valerie B. Duffy

**Affiliations:** 1 Department of Allied Health Sciences, University of CT, Storrs, CT 06269, USA; kate.killion@uconn.edu (K.E.K.); amy.corcoran@uconn.edu (A.C.); laura.gilbert@uconn.edu (L.G.); 2 Nutrition and Dietetics, Doisy College of Health Sciences, Saint Louis University, St. Louis, MO 63104, USA; maria.romopalafox@health.slu.edu; 3 Rudd Center for Food Policy and Health, University of CT, 1 Constitution Plaza, Suite 600, Hartford, CT 06103, USA; jennifer.harris@uconn.edu; 4 Walden Behavioral Care, Dedham, MA 02026, USA; inna.kagan@uconn.edu

**Keywords:** caregivers, feeding practices, children, responsive feeding, mixed method

## Abstract

Responsive feeding (RF), the reciprocal feeding approach between caregiver and child that promotes child health, is understudied among low-income caregivers. This mixed methods study with low-income caregivers of 12-to-36-month-olds aimed to (1) assess variability in RF and associations with children’s dietary intake, and (2) explore caregivers’ perceptions of RF. Caregivers (*n* = 134) completed an online survey with RF questions (*n* = 25), grouped into environmental (meal environment, caregiver modeling, caregiver beliefs) and child (self-regulation, hunger/satiety cues, food for reward, food acceptance) influences scores. Children’s recent food group consumption was loaded onto healthy and less healthy intake scores. In an adjusted multiple linear regression analysis, greater RF scores for environmental and child influences were associated with greater healthy intake scores (*p*’s < 0.01). Greater scores for environmental influences were also associated with lower scores for unhealthy intake (*p* < 0.01). From focus groups with a separate sample of caregivers (*n* = 24), thematic analysis uncovered that two themes aligned (trust in child cues, positive strategies to encourage children to eat non-preferred foods) and two misaligned (lack of trust in child cues, use of force/bribery) with RF. Complementary integration of quantitative and qualitative findings can inform future interventions with low-income caregivers, encouraging trust in young children’s hunger/satiety cues and positive strategies for food acceptance to improve diet quality.

## 1. Introduction

Young children and toddlers in the United States (U.S.) and other high-income countries are at risk for poor diet quality, with excess consumption of refined grains, added sugars, and sodium, and inadequate intake of vegetables and whole grains [1,2,3,4,5,6,7]. Although the 2009–2014 data from the National Health and Nutrition Examination Survey (NHANES) show that young children above and below the federal poverty threshold have poor diet quality [2], those who are racial and ethnic minorities and from low-income families and neighborhoods have the highest risk of poor diet quality [2,8]. There are multi-level spheres of influence on a young child’s dietary intake [9], including the interactions between innate child characteristics and the parent’s/caregiver’s role in the provision of food and their style of feeding their child.

Responsive feeding (RF) is a feeding style characterized by child signaling of hunger and fullness cues and the appropriate responses from the caregiver to these cues [10]. Its definition has grown into a comprehensive, multi-dimensional framework of parent/child interactions and responses that are influenced by a range of demographic, environmental, and child-related conditions. Optimally, RF involves the provision of nutrient-dense foods to support high diet quality, structured and predictable eating times, and a pleasant eating environment with family interactions and minimal distractions [10]. Expert consensus is that non-RF behaviors, such as using food for behavior management or controlling the child’s intake, may foster the development of poor diet quality and excessive energy intakes through interfering with the child’s autonomy and by overriding hunger and satiety cues [10,11]. In contrast, RF may improve diet quality by promoting the child’s development of preferences for healthy foods [10,11].

Various dimensions of RF practices are associated with toddler food preferences and diet quality. For example, controlling feeding practices may reduce child acceptance and intake of vegetables [12], whereas repeated exposure, modeling, and use of non-food rewards may increase child liking and intake of vegetables [13]. However, few studies have assessed the relationship between diet quality and RF as a comprehensive, multi-dimensional feeding practice. In an online survey study with a large sample of Australian mothers, a latent construct of lower non-responsive feeding practices (reward for behavior, reward for eating, persuasive feeding, overt restriction) and higher structure-related feeding practices (covert restriction, structured meal setting, structured meal timing, monitoring, modeling) was associated with greater diet quality among their 2–5-year-old children [14]. Additional studies assessing the relationship between comprehensive RF practices and diet quality are needed.

A research priority has been identified to improve the understanding of how to implement RF-related recommendations across households with different income levels within high-income countries [15]. Such work is critical, given that barriers and facilitators of practicing RF to achieve healthier diets likely differ among low-income families compared to high-income families, impacting the potential success of RF interventions. For instance, energy-dense food may be used to protect against feelings of food insecurity among families with low income, thus increasing child preference for these foods [16,17,18,19,20]. Characteristics of lower-income caregivers may hinder optimal RF practice, including lower nutrition-related knowledge [21], time constraints that interfere with meal planning and family mealtimes [22], and psychosocial goals that conflict with healthy eating goals [23]. Additionally, disruptive life events related to low income—such as unstable employment, residence, and childcare—pose additional practical barriers to healthy dietary patterns [24]. Families with low incomes also may be exposed to high rates of targeted marketing for unhealthy toddler drinks and snacks, which could influence the RF relationship [25,26,27,28]. These demographic and environmental conditions could influence the variability of RF practices among families and the ability for RF to positively influence child diet quality.

Thus, this mixed methods study sought to explore RF beliefs and practices among caregivers of 12-to-36-month-old children sampled from a single low-income community. First, we aimed to assess variability in survey-reported RF beliefs and practices among caregiver/child dyads and test relationships between RF and measures of the child’s dietary intake. Second, to provide additional context, we conducted focus groups to obtain a deeper understanding of beliefs and behaviors about RF among low-income caregivers. Lastly, we aimed to integrate the quantitative and qualitative results. These findings will help inform future interventions that promote RF practices as a means to improve diet quality among low-income caregivers with young children.

## 2. Materials and Methods

### 2.1. Study Design

**Figure 1:**
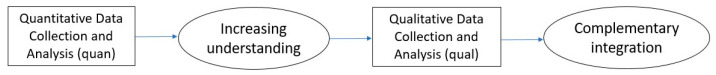
Depiction of sequential explanatory mixed methods design.

This paper reports on the RF component of a mixed methods study examining beliefs and behaviors related to healthy beverage and snack provision as well as RF in low-income families with young children. The qualitative findings on healthy beverages and snacks have been published elsewhere [29]. Mixed methods, an increasingly important research approach, aims to better understand a phenomenon through integration of quantitative and qualitative findings [30,31]. We used an explanatory sequential mixed methods design (Figure 1), in which qualitative findings (focus groups) complemented quantitative findings (online child feeding survey) by providing greater insights into the caregiver’s understanding of RF recommendations, experiences, and barriers to implementation [32]. In this design, data are interpreted together [32].

In the quantitative phase, caregivers of children aged 12–36 months completed a one-time, online survey of toddler feeding attitudes and practices, generated by the research team according to the Healthy Eating Research (HER) guidelines and the 2020–2025 Dietary Guidelines for Americans [33,34]. In the qualitative phase, caregivers of 12- to 36-month-old children participated in a 90 min focus group, which sought to provide greater insight into RF practices and barriers to RF. Methods for each phase are described below.

### 2.2. Survey Procedures

The online survey was created by a team of experts in dietetics, toddler nutrition, food marketing, and survey development. The survey was developed to be consistent with the Healthy Eating Research (HER) guidelines [34] and the 2020–2025 Dietary Guidelines for Americans [33]. Following survey development, the content was validated by experts in food marketing, pediatric nutrition, developmental psychology, public health, social work, and social media [35]. The survey was pilot tested among 15 parents of young children, adapted to reduce participant burden, and to promote survey understanding. The final survey was 94 questions including RF; breastfeeding; introduction of complementary foods and the child’s food/beverage intake; parental attitudes and beliefs including those about developing healthy food preferences; feeding context; food acculturation; food security; and trusted sources of information and preferred communication channels. 

A convenience sample of caregivers of young children was recruited in a single town in Connecticut outside of the capital city in 2018–2019. At the time of data collection, about 30% and 40% of residents in the town identified as Black/African American or Hispanic/Latino, respectively [36]. About 22% of residents received federal nutrition assistance (Supplemental Nutrition Assistance Program), and ≥50% of the school children were eligible to receive reduced or free school meals [29]. Caregivers were recruited via flyers distributed at community organizations serving families with low income (community health centers, Supplemental Nutrition Assistance Program for Women, Infants, and Children [WIC], family resource centers, childcare centers). The flyers described the online survey, and researchers were available on-site to answer questions and determine participant eligibility. 

Inclusion criteria included the following: (1) being a primary caregiver of at least one child between 12 and 36 months and (2) being one of the primary food providers and decision makers for the child. Individuals were excluded from the study if (1) their child was on a special diet due to an error of metabolism or another nutrition-related disorder, or (2) they could not complete the survey in English or Spanish. Caregivers provided informed consent as the first question in the online survey. All participants who completed the survey received a $10 gift card and nutrition education materials (e.g., toddler feeding handouts, memo pads, pencils) as incentives. The University of Connecticut Institutional Review Board approved the online survey (#X17-175).

Caregivers completed the online survey on-site using tablets. Surveys were hosted on a secure server through the University (Qualtrics, Provo, UT). The survey began with household demographics, including the total number of children in the home and the number of children between 12 and 36 months. If the caregiver reported having more than one child aged 12–36 months, they were asked to choose one child to report information on for the remaining survey questions. The survey also asked for participant demographics, including age of the child and caregiver (in months and years, respectively); gender, race, and Hispanic/Latino ethnicity; caregiver education; history of breastfeeding the child (formula only, formula and breastmilk, or breastmilk only); and household food security status. Individuals were considered food insecure if they answered “often true” or “sometimes true” to either question on the 2-item food insecurity screener [37]. The median time to complete the final survey was 28 min.

#### 2.2.1. Responsive Feeding and Conceptual Constructs of Environmental Influences and Child Influences

Twenty-five of the survey questions fit conceptually into the components of a comprehensive, multi-dimensional, and theoretical [38] framework of RF as shown in Table 1. Questions had a variety of response options (Supplementary Materials) that were scored between 0 and 4, with higher scores more aligned with RF. For higher scores to be more aligned with RF, some items had to be reverse coded. Scores for the 25 questions were placed into seven conceptual groups and averaged, for a score of 0 to 4 for each conceptual group. 

Next, the seven RF conceptual groups were placed into exploratory principal component analysis (PCA) to identify factors based on factor loading ≥ 0.4. The conceptual groups formed two factors that explained 42% of the variance across all responses (Supplementary Materials), supporting the multidimensionality of the conceptual groups. Furthermore, the factors could be conceptually labeled as (1) Environmental Influences, including the Meal Environment, Food Offered and Caregiver Modeling, and Caregiver Beliefs about Healthy Preferences, and (2) Child Influences, including Child Self-regulation, Child Hunger and Satiety Cues, Food for Reward or Behavior, and Child Food Acceptance (Table 1). The separate factors had poor internal reliability as tested with Cronbach’s alpha (~0.4). However, given the two-factor structure of the PCA and the complexity of RF, the alpha statistic may underestimate reliability [39]. The two RF factor scores were the sum of all variables in each factor and dividing by the number of conceptual groups. Thus, each RF score was valued between 0 and 4, with higher scores indicating greater alignment with responsive feeding.

#### 2.2.2. Intake Patterns for Food Groups to Create Healthy and Less Healthy Scores

Caregivers were asked two types of questions about their child’s dietary intake within the last week. These measures were selected over full-length food frequency questionnaires or 24 h dietary recalls to reduce the average time it would take participants to complete the survey. One set of questions assessed the frequency of food groups consumed in the past 7 days by frequency category and scored as frequency per week (never = 0, 1–2 days = 1.5, 3–4 days = 3.5, 5–6 days = 5.5, everyday = 7). The food groups were fruits (but not pureed in pouches or jars), vegetables (but not pureed in pouches or jars), 100% fruit or vegetable juice, sugar-sweetened beverages, snacks (plain cereal, sweets, crackers, fruit snacks, baby/toddler snacks, sweetened cereals, salty snacks), and fast food. For each group, the online survey showed a picture of the foods/beverages in the group to clarify the meaning. The second set of questions asked caregivers to report diversity of foods consumed within each food group. Such questions were included to reflect the Dietary Guidelines for Americans’ recommendation for children to consume a variety of foods in each MyPlate food group. Within each food group, caregivers were asked to select the foods and beverages the child consumes. The caregiver could select (yes/no) up to 9 types of fruits, 11 types of vegetables, 7 types of sugar-sweetened beverages, and 7 types of snacks. Total selected (yes) responses were summed to provide a diversity score for each group.

These diet variables (frequency and diversity scores for each food group) underwent exploratory PCA to identify factors based on factor loading ≥0.4. We identified two dietary intake factors which explained >50% of variability and could be labeled as “less healthy intake” (days of juice, fast food, and sweet consumption; diversity of sweet drinks and snack foods consumed; alpha = 0.61) and “healthy intake” (days of fruit and vegetable consumption; diversity of fruit and vegetable consumption; alpha = 0.67). Less healthy and healthy intake scores were created by summing the variables included in each respective factor. Initial scores could thus range from 0 to 35 for less healthy and 0 to 34 for healthy intake patterns. For both healthy and less healthy intake scores, higher scores indicate greater frequency and diversity of intake.

### 2.3. Focus Group Procedures

For phase two, we conducted in-person focus groups with a convenience sample of caregivers recruited in the same low-income community from March to June 2019. Caregivers were invited to participate via flyers distributed at community organizations serving families with low incomes (community health centers, WIC, family resource centers, and childcare centers). The flyers described the purpose of the study and provided a sign-up sheet for focus groups. The research personnel also visited locations to provide information about the study to potential participants in person. The researchers contacted caregivers interested in the focus groups to describe the study and determine eligibility to participate. 

Inclusion criteria included the following: (1) being a primary caregiver of at least one child between 12 and 36 months and (2) being one of the primary food providers and decision makers for the child. Individuals were excluded from the study if (1) their child was on a special diet due to an error of metabolism or another nutrition-related disorder, or (2) they could not complete the focus group in English or Spanish.

Caregivers participated in one of seven focus groups conducted in English (6 groups) or Spanish (1 group). All study materials were translated into Spanish by a post-doctoral researcher, registered dietitian, and native Spanish-speaker (MRP). Each focus group included 2–6 participants. Sessions were conducted over 90 min at WIC offices and public libraries. On-site childcare was provided for participants if required. A member of the study team trained in focus group facilitation (JH) and a native Spanish-speaker (MRP) moderated the groups. A notetaker was present at each session, and the sessions were audio-recorded. After signing informed consent forms, participants introduced themselves and shared information about their child’s age, their role in feeding their child, and any challenges they have experienced in feeding their toddler. 

The moderator led a table discussion using visual handouts, including two handouts depicting RF guidelines (Supplementary Materials). Additional handouts referenced healthy beverages and snacks (results previously published). The two RF topics, portion size and hunger and satiety cues, were identified as areas of concern in the online survey. After presenting each handout, the moderator asked how the caregivers/parents felt about the guideline, whether it was realistic, what would make it hard to follow the guideline, and other related questions. Data saturation was reached in groups six and seven, and researchers determined that no additional groups were necessary.

Caregivers provided informed consent at the beginning of each focus group. All participants who completed a focus group received a $20 gift card as incentive. The University’s IRB approved all study procedures (#X19-009).

### 2.4. Data Analysis

For phase one (survey), all statistics were performed using SPSS Statistics 25 (SPSS Inc., Chicago, IL, USA). Descriptive statistics were used to summarize characteristics of survey participants, including caregiver/parent and child age, caregiver/parent and child race/ethnicity, caregiver/parent and child gender, household food security, and caregiver/parent education. Descriptive statistics summarized the frequency of consuming snacks, juice, sweet drinks, fruit, and vegetables. We first compared food group frequency with approximate recommendations in the Dietary Guidelines for Americans [33] and HER guidelines [34], calculating the percent of children who approached the approximate guidelines. Dietary Guidelines recommend daily consumption of fruit and vegetables, avoidance of all sugar-sweetened beverages and limitation of added sugar to <10% of total calories for children over 2 years, and consumption of juice up to 4 oz per day [33]. While there are no specific recommendations for snacks, the U.S. Dietary Guidelines for Americans recommend limitation of high sodium foods, including salty snacks. HER guidelines encourage consumption of healthy snacks with at least two food groups [34]. We identified children as approaching these guidelines using the following criteria for each food group: fruit (daily), vegetables (daily), juice (infrequent, 1–2 times/week or less), sweetened beverages (none), snack foods (none), and fast food (none). 

Next, we used descriptive statistics and the F test for equality of variances to describe the variability in RF scores and conceptual groups. Then, we used multiple regression analyses to test the relationship between RF and intake factors. All variables were assessed for normality using the Shapiro–Wilk or Kolmogrov–Smirnov test, where a *p* < 0.05 indicated non-normal distribution. The less healthy intake score was transformed via square root due to non-normal distribution. All linear regression models were adjusted for child age, history of breastfeeding (coded as 0 = formula only, 1 = formula and breastfeeding, 2 = breastfeeding), and household food security status (food secure versus food insecure) to reflect previous literature suggesting that these variables may influence child diet quality [40,41,42], checking for collinearity and multivariate outliers [43].

The analysis of the focus groups was conducted independently of the quantitative findings. Focus audio-recordings were transcribed verbatim. The Spanish session was transcribed and translated to English prior to data analysis. Transcripts were analyzed using Thematic Analysis [44]. First, researchers familiarized themselves with the data by reading transcripts. Next, researchers generated initial codes based on the topics discussed in the focus groups. Three research assistants independently coded the transcripts using NVivo 12 and Microsoft Excel (version 2401). Meetings occurred between the moderator and coders to refine codebook definitions until consensus was reached. Qualitative reliability testing was conducted during group meetings to ensure coders were using the codebook equivalently. After coding was complete, themes were developed and refined by the study team. This paper presents themes related to RF. 

Lastly, the results are presented in an integrated results matrix, which shows qualitative and quantitative results side by side [31]. The side-by-side comparison was designed for the purpose of complementarity, or the use of one method to enhance or illustrate the results of another method [31]. Exemplary quotes were selected which illustrated both quantitative results and qualitative themes.

## 3. Results

### 3.1. Survey Results

#### 3.1.1. Participant Characteristics

In total, 134 caregivers (93% female) participated in the survey. Caregivers had an average of 2.31 (±1.24 SD) total children, and 1.10 (±0.37) children who were ages 12–36 months. The average age of sampled children was 22.8 (±7.3) months. The sample was skewed towards families with low socioeconomic status, with 26% screening positive for food insecurity and 83% receiving WIC benefits, as well as 74% completing less than a 4-year college degree. About two-thirds of caregivers and children were each from an underrepresented racial/ethnic group. Twenty-five percent of the children were only fed formula, 46% formula and breastmilk, and 28% only fed breastmilk. Participant characteristics are presented in Table 2. 

#### 3.1.2. Intake Patterns Compared to Guidelines for Healthy Eating

Caregiver-reported child dietary intake deviated from recommended intake levels. Approximately half of children consumed fruit (58%) and vegetables (46%) at least daily for the prior week. And over half (58%) of the children consumed juice 3–4 times per week or more and sweetened beverages (55%) at least once in the past week. Nearly all (97%) children consumed snack foods at least in the past week. Table 3 shows reported food group intake aligned with recommendations in the Dietary Guidelines for Americans and HER guidelines is shown in.

#### 3.1.3. RF Variability and Association with Children’s Dietary Intake

The environmental influences score averaged 2.8 of 4 points possible (SD = 0.4, range = 1.9–3.7) and was normally distributed (Kolmogrov–Smirnov = 0.06, *p* = 0.08). Of the conceptual groups within this score, meal environment had the highest mean (3.3, range = 1.4–4), the caregiver nutrition beliefs had the lowest mean (2.0, range = 1.0–3.5), and the food offered and caregiver modeling fell in between (2.9, range = 1.3–4.0). The scores had similar variability (variances between 0.3 and 0.4).

The child influences score averaged 2.5 of 4 points possible (SD = 0.4, range = 1.3–3.5) and was normally distributed (Kolmogrov–Smirnov = 0.05, *p* = 0.57). Of the conceptual groups within this score, child hunger and satiety cues had the highest mean (2.9, range = 1.0–4.0), followed by child food acceptance (2.8, range = 0.5–4.0), child self-regulation of intake (2.3, range = 0.0–4.0), and food for reward or behavior (2.1, range = 0.0 = 3.7). Scores had high to moderate variability (variances between 0.4 and 0.9), with the food acceptance conceptual group having a significantly greater variance than the food for reward or behavior and child hunger and satiety scores groups (F test of equality of variances = 0.6, *p’s* < 0.01).

**Figure 2:**
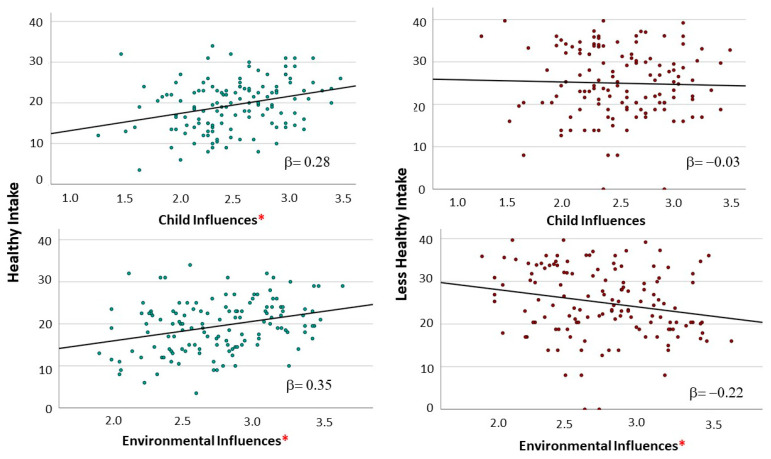
Scatterplot depicting association between dietary intake patterns and responsive feeding scores. * Responsive feeding scores with significant association with intake scores; *p* < 0.01.

Separate multiple linear regression analyses were conducted to predict dietary intake scores (healthy and less healthy) from the RF scores (environmental influences and child influences) while adjusting for the child’s age, history of breastfeeding, and household food insecurity. Bivariate associations between the RF factors and the children’s dietary intake are shown in Figure 2. Higher environmental influences scores were associated with lower scores for less healthy intake (β = −0.22, *p* < 0.01) and separate from significant effects of greater age (β = 0.32, *p* < 0.01), and non-significant effects of breastfeeding (β = −0.06, *p* = 0.47) and food insecurity (β = 0.06, *p* = 0.51). In addition, higher environmental influences scores were associated with higher scores for healthy intake (β = 0.35, *p* < 0.01) and separate from significant effects of breastfeeding (β = 0.23, *p* < 0.01), and non-significant effects of the child’s age (β = −0.10, *p* = 0.26) and food insecurity (β = 0.15, *p* = 0.08) Higher child influences scores were associated with higher scores for healthy intake (β = 0.28, *p* < 0.01) and separate from significant effects of breastfeeding (β = 0.18, *p* < 0.05) and non-significant effects of the child’s age (β = −0.06, *p* = 0.51) and food insecurity (β = 0.09, *p* = 0.29). There was a flat relationship between the child influences score and less healthy intake score (β = −0.03, *p* = 0.98) with significant effects of greater age on high scores for less healthy intake (β = 0.30, *p* < 0.01) and additional non-significant effects of breastfeeding (β = −0.05, *p* = 0.52) and food insecurity (β = 0.12, *p* = 0.19). 

### 3.2. Focus Group Results

#### 3.2.1. Participants

A total of 24 adults (23 female, 1 male) participated across seven focus groups. There were 21 parents, 2 grandparents, and 1 aunt among the participants. Among those who self-identified their race/ethnicity during the focus group, seven were Black, eight were Hispanic/Latino, and two were Asian.

#### 3.2.2. Focus Group Themes

The thematic analysis identified four themes related to RF: (1) Lack of trust in child fullness cues, (2) Trust in child fullness cues, (3) Using force and bribery to ensure children eat enough and a variety of foods, and (4) RF-aligned strategies to encourage children to eat non-preferred foods. Table 4 presents sample quotes for each theme. Each theme is discussed in depth below. 

##### Lack of Trust in Child Hunger or Fullness Cues

Caregivers expressed concern that their children were eating “too much” or “not enough.” Those who felt that their child ate too much reported that their child would continue eating despite visible abdominal distention or onset of verbalized physical discomfort (e.g., stomachache). This lack of demonstrated satiety was often attributed to distraction, including toddlers using screens during mealtime. Other times, caregivers expressed that their child was not eating adequately at school and therefore engaging in compensatory overeating when they got home.

Other caregivers felt that they knew what an appropriate amount of food for a toddler was, yet that their child ate less than this amount. These caregivers reported that their children preferred to forgo food to access television or play. Some caregivers were concerned that their children were wasting food and believed that children should eat what is served.

##### Trust in Child Hunger or Fullness Cues 

Conversely, some, albeit fewer, caregivers shared that they allow their toddler to decide how much and/or when to eat through demonstration of age-appropriate hunger and satiety cues. These include spoken (e.g., stating they are full) and physical (e.g., pushing the plate away) demonstration of the child’s desire to stop eating. Several parents shared that they responded to these cues by offering an initial quantity to the child, and then providing subsequent servings if requested by the child. Several parents described following these cues to decide when to provide food to the child (e.g., providing a snack when the child states that they are hungry). A small number of parents reported that children should determine intake due to a belief that “their body knows best,” and due to advice from professionals such as pediatricians and daycare teachers.

##### Using Force and Bribery to Ensure Children Eat Enough and a Variety of Foods

Caregivers frequently reported that their children preferred fruit and processed “snack foods” over meat and vegetables. To combat these preferences, caregivers often required that their children finish non-preferred foods when they were offered at meals. Often, caregivers would leverage their child’s internal drive to eat sweet food, only allowing the child sweets after they consumed a non-preferred food. Other caregivers reported using non-food rewards such as screen time to encourage eating. 

##### RF-Aligned Strategies to Encourage Children to Eat Non-Preferred Foods

Caregivers also reported various RF-aligned strategies to encourage children to try new foods. Reported strategies included offering a variety of foods, creating a structured feeding schedule, serving both preferred and non-preferred foods, and serving non-preferred foods in a new way. Many caregivers also recognized the need to introduce foods multiple times.

### 3.3. Integration of Quantitative and Qualitative Findings

Combined quantitative and qualitative results are shown in Table 5. The association between the child influences score with the healthy intake score is illustrated by two focus group themes: trust and lack of trust in child cues. Caregivers who did trust child cues described scenarios in which children self-regulated their intake of both nutrient-dense and nutrient-poor foods. In contrast, caregivers who expressed a lack of trust in child cues often described behaviors that could contribute to lower intake and diversity of fruits and vegetables. For example, caregivers described forcing the child to eat vegetables, which could inadvertently lead the child to develop less preference for vegetables. 

The association between environmental influences and healthy intake scores is illustrated by focus group reports of positive strategies to encourage nutrient-dense food intake. Caregivers often described responsive-feeding-aligned external strategies to encourage fruit and vegetable intake, such as incorporating vegetables into preferred dishes. In contrast, the negative association between the environmental influences score and less healthy intake score is illustrated by caregiver reports of force and bribery. Often, caregivers reported using less healthy foods (e.g., sweets) as a reward. This practice is non-responsive in nature and may directly increase intake of foods included in the less healthy score.

## 4. Discussion

The purpose of this mixed method study was to examine responsive feeding practices and beliefs among low-income caregivers of children aged 1–3 years and the potential effects on children’s dietary intake. From an online survey, the RF practices fell into two conceptual groups, environmental influences and child influences, that showed good variability and normal distribution. The environmental influences factor captured the mealtime environment, caregiver beliefs and attitudes, and caregiver modeling. The child influences captured child hunger/satiety cues, child self-regulation, food for reward, and food acceptance, the latter of which showed the greatest variance. Accordingly, the caregivers reported variability in diet intake of the children, including poor patterns of dietary intake, suggesting the high need for nutrition intervention. The environmental and child influences scores were significantly associated with healthier intake patterns. Specifically, the environmental influences RF score was associated with less unhealthy dietary intake patterns and with more healthy intake patterns. A higher child influences score, reflective of child-driven food regulation, less use of food for behavioral control, and greater healthy food acceptance, was positively associated with healthy intake. In focus groups, caregivers discussed child behavior (e.g., display of hunger and fullness) as well as environmental influences scores that may prompt the child to accept or reject food. Such practices could inadvertently impact diet quality. 

Our findings are positioned within a substantial body of RF literature. However, our work fills a crucial need to assess the relevance of RF among families with low incomes, and the association between RF and diet quality among this demographic group. This work may be used to better the understanding of RF and responsive parenting frameworks among scholars working with low-income families [15]. Existing interventions to improve RF practices in low-income caregivers with young children have been pilot-tested [45,46] and can support increases in RF behaviors [46,47], with potential to improve diet quality [46] and decrease the child’s risk of overweight/obesity [45]. However, given that most observational studies of barriers to RF have been conducted among families with higher incomes [48], these and other interventions could be tailored to reflect the greater understanding of RF practices among families with low incomes generated in our work. 

For example, our results may inform the creation of tailored nutrition communications. Such strategies are needed among families who report receiving conflicting nutrition advice [25,26] and are exposed to targeted unhealthy snack and beverage marketing [27,28,49]. In the future, tailored nutrition education messages can be incorporated into digital interventions among low-income children and caregivers [50]. Messages related to RF, including those presenting information about child control of intake, are acceptable among caregivers of young children [51], though message development and testing among more diverse families is needed [51]. Such messages could be delivered through existing federal nutrition education programs that reach families with low incomes, including the Supplemental Nutrition Assistance Program-Education (SNAP-Ed) and the Supplemental Nutrition Assistance Program for Women, Infants, and Children (WIC).

Our survey and focus group results may be understood in the context of biological, social and cultural, and environmental determinants of RF and toddler diet quality. First, focus group participants commonly discussed that children dislike new (neophobia) or specific (selective or “picky” eating) foods. Similarly, child selective eating and neophobia were included in the child influences score which is associated with a higher intake/diversity of fruits and vegetables. The food acceptance score was highly variable, which may reflect variability in innate child taste preferences. Food refusal is common among infants and toddlers, particularly when providing vegetables with bitter or unfamiliar flavors [52]. Although young children develop liking with repeated exposure to healthy foods [52], consistent food refusal can be emotionally taxing and lead parents to present preferred foods instead of more nutrient-dense “challenge foods” [53,54]. Additionally, caregivers’ emotional reactions to children’s food refusals—such as feelings of frustration and anger—may increase the child’s negative association with such foods [55]. Caregivers from low-income families may also choose to provide preferred foods over non-preferred foods to reduce waste, potentially increasing picky eating risk [56]. Thus, children with low food acceptance may fail to meet recommendations to eat a variety of fruits and vegetables [33].

Additionally, trust in child hunger and satiety cues were frequently discussed among focus group participants, highlighting the need to clarify uncertainty among families with low incomes. While there is biological variance in hunger drives among children, infants and toddlers are largely able to self-regulate their caloric intake to maintain healthy weight [57]. Given that energy-regulating mechanisms diminish as the child ages [58], encouraging RF among low-income families at an early age is needed. Low income may present barriers to caregiver trust in child hunger and satiety cues through influencing food parenting practices and child eating behaviors. 

Social and cultural factors may influence the low-income dyad’s ability to engage in RF and promote healthy diet quality. Attempting to control child intake, either through pressure or restriction, may increase intake of unhealthy foods, dislike of healthy foods, and eventual overweight and obesity [59,60]. However, our focus group participants commonly reported pressuring children to eat certain types or amounts of food or restricting the intake of these foods. These practices are common among low-income caregivers outside of the present analysis. For example, in an analysis of low-income Latina mothers participating in the New York City WIC program, most mothers exhibited a pressuring feeding style, despite 93% of participants believing infants can determine satiety [61]. One potential explanation for why parents assume this control is that “parents know best” and act based on their perceived parental instincts, as shared in a previous qualitative study of WIC families [62]. Alternative explanations may relate to the need to reduce food waste or save time [53] and negative parental experiences with low food access [63]. Such practices should be discouraged because children’s diminishing ability to regulate their energy intake is due in part to external environments influencing intake amount and quality [58]. 

Additionally, in both the survey and focus groups, caregivers frequently reported using food as a behavior control mechanism, which conflicts with RF recommendations and may warrant nutrition messaging. Caregivers commonly use less healthy foods and snacks to manage a child’s behavior, either to prevent “bad” behavior or to reward “good” behavior [64,65]. Such approaches may promote emotional eating and diminish the child’s ability to self-regulate energy intake [66,67]. Future interventions should assess and address food as a behavior management tool, including solutions for evidence-based behavior management that may be applicable to families with low incomes. Such interventions may use the Feeding to Manage Child Behavior Questionnaire developed among low-income families with preschool-aged children [64]. 

Caregiver/parent nutrition beliefs are also an important social and cultural influence of RF and diet quality. In an optimal RF relationship, the food provided should primarily align with the Dietary Guidelines for Americans [10,33]. In our feeding survey, caregiver beliefs about the need for special foods and the importance of fruits and vegetables contributed to the environmental influences score, which was associated with child intake of health-promoting and less health-promoting foods. Future interventions to improve RF and diet quality may clarify common misbeliefs about child nutrition.

Lastly, the food environment is critical to RF and may positively influence diet quality. During RF, caregivers are encouraged to create a pleasant meal environment where the child is face-to-face with other family members [10]. Meals should follow a predictable schedule. and distractions should be minimized [10]. In our sample, the meal environment was included in the environmental influences score and represents eating as a family, eating at the table, screen access, and concordance between child food and family food. A large body of literature supports the influence of the meal environment on diet quality. For example, family meals are associated with healthier food intake [68], while alternative meal environments, such as eating in front of the TV, are associated with higher BMI, increased energy intake, and unhealthful diets [69,70,71,72,73,74,75]. However, encouraging family meals among low-income families may be challenged by busy work schedules [76]. Interventions that support a RF-aligned meal environment may be warranted among families with low incomes.

### Limitations and Strengths

This study has several strengths. First, the participant survey was created and its content validated by a team of experts in nutrition, food marketing, and survey design. Expert consensus concluded that survey measures, including RF and diet quality questions, were satisfactory. Further, we measured RF using a broad range of concepts, including infant/toddler cues, parent responses, meal environment, and parent nutrition knowledge. While our questionnaire has not been validated, it comprehensively assessed RF, unlike existing validated tools [77]. Next, our diet quality assessment captured dietary diversity to reflect the Dietary Guidelines for Americans’ recommendation for children to consume a variety of healthy foods in each food group [33]. Additionally, our integration of both qualitative and quantitative findings provided deeper understanding of participant experiences than either method could alone. Finally, our sample was racially and ethnically diverse, thus increasing the generalizability of our results.

Several limitations should be considered when interpreting the present study. First, the survey was cross-sectional, and the focus group results were qualitative. Thus, causality or directionality of the association between RF and diet quality cannot be determined. Next, the findings from the focus groups were not member-checked and were subject to the biases of the researchers, including bias related to the researchers’ lived experiences and socioeconomic status. The RF index and conceptual group scores were also exploratory and have not been validated, and the limited internal reliability of the RF factors (external influences, child influences) suggests they did not reflect a unitary construct. However, there is currently no widely accepted, comprehensive tool to assess RF, though recommendations to create such a tool have been made [77,78,79]. Validation of tools among historically marginalized groups is needed [77]. Similarly, due to concerns about survey length, we did not use a validated, full-length food frequency questionnaire or 24 h recall to assess food intake, which may lead to recall and social desirability biases. Future research should assess the relationship between RF and diet intake among a diverse population using a validated food intake measure. Additionally, as we recruited a convenience sample of participants with low incomes, results may not be generalizable to a wider population. Next, though all caregivers were recruited in the same community, different samples were used for the quantitative and qualitative study phases, in contrast to the typical explanatory sequential mixed method design [32]. Lastly, our findings may not be generalizable to caregivers living in low- and middle-income countries. Future research may continue to explore the relationship between RF and undernutrition in these settings [80]. 

## 5. Conclusions

We found that responsive feeding (RF) was a relevant concern among families with low incomes and was associated with proxies of toddler diet quality. This work fills a critical gap in the literature to better understand RF and its association with diet quality among families with low incomes. In this mixed methods paper, we identified two RF factors: environmental influences (meal environment, caregiver beliefs and modeling) and child influences (child hunger/satiety cues, child self-regulation, food for reward, food acceptance). Positive caregiver influence scores were associated with greater children’s intake of fruits and vegetables and decreased intake of less nutrient-dense foods (snacks, sweets, juice, fast food). Positive child influences scores were associated with greater children’s intake of fruits and vegetables. Future interventions may aim to shift modifiable environmental influences to improve low-income toddler diet quality, such as encouraging the use of fruit as sweets, promoting parental modeling of healthy eating, and avoiding the use of food as a behavior management tool. Future interventions may also encourage caregiver trust in child hunger and fullness cues. Lastly, future high-quality research and nutrition communication focused on RF among diverse families with low income are needed to support efforts to improve toddler diet quality.

## Figures and Tables

**Table 1:** A total of 25 questions about responsive feeding were placed in conceptual groups and categorized into two RF factors (environmental influences, child influences).

Conceptual Group	Survey Questions ^1^
Environmental Influences	
Meal Environment	My child ate with the rest of the family My child ate at the table or in a high chair My child ate while watching TV My child ate on the go, such as in a stroller or car seat, or on the bus I enjoy spending time with my child at mealtimes
Food Offered and Caregiver Modeling	Other people in my family make it hard for me to feed my child healthy The food we eat as a family provides enough nutrition for my child
Caregiver Nutrition Beliefs	Picky eaters need products like Pediasure^® 2^ Enfagrow^® 3^, or Nido^® 4^ to get enough nutrition Toddler formulas or powdered milks provide nutrition that children don’t get from other food and drinks Pureed food that comes in pouches is a good way to teach toddlers to like the taste of fruits and vegetables Children won’t eat the same food as the rest of the family. They need their own type of food 100% juice is a good choice if a child won’t eat fruit or vegetables Children should be served fruits and vegetables every day
**Child Influences**	
Child Self-Regulation of Intake	[Picture of a meal shown] Is it like the amount of food you serve at a meal for your child? Who decides how much food your child eats? Do you make your child finish all the food you serve?
Child Hunger and Satiety Cues	My child tells me when he or she is hungry I worry that my child eats too much It is difficult to get my child to eat enough at meals
Food for Reward or Behavior	It’s OK to give children drinks with added sugar once in a while It would be mean to not give children sweet treats once in a while In the past 7 days, how many days did you give your child something to eat or drink because he or she was fussy?
Child Food Acceptance	My child only eats a few foods My child will not taste a new food

^1^ Responses to all questions scored from 0 to 4, with higher scores indicating greater alignment with responsive feeding recommendations. Responses to questions aligned versus not aligned with responsive feeding were scored in opposing directions (e.g., 0 to 4 or 4 to 0). ^2^ Abbott Nutritional Products, Abbott Park, IL, USA; ^3^ Nestlé, Arlington, VA, USA; ^4^ Mead Johnson Nutrition, Chicago, IL, USA.

**Table 2:** Household, caregiver, and child demographics reported in an online toddler feeding survey of low-income caregivers.

	*n* (%)
***Household***	
**Number of children in home**	
1	41 (30.6)
2	44 (32.8)
3	24 (17.9)
4	19 (14.2)
5+	6 (4.5)
**Number of children between 12 and 36 months**	
1	123 (91.8)
2+	11 (8.2)
**Food security**	
Food Secure	99 (73.9)
Food Insecure	35 (26.1)
**Participation in food assistance programs**	
WIC	111 (82.8)
SNAP	66 (49.3)
Mobile food pantry	14 (10.5)
Food pantry	22 (16.4)
***Caregiver***	
**Age (years)**	
18–24	16 (11.9)
25–34	78 (58.2)
35–44	33 (24.7)
45+	7 (5.2)
**Gender**	
Male	10 (7.5)
Female	124 (92.5)
Other	0 (0)
**Race/ethnicity**	
White	26 (19.4)
Black/African American	36 (26.9)
Hispanic/Latino	43 (32.1)
American Indian/Alaska Native	1 (0.7)
Asian/Pacific Islander	5 (3.7)
More than one race/ethnicity	19 (14.2)
Other	4 (3.0)
**Education**	
Less than high school	10 (7.4)
High school/GED	35 (26.1)
Some college or trade school	37 (27.6)
2-year college	17 (12.7)
4-year college	23 (17.2)
Master’s degree or higher	12 (9.0)
***Sampled Child 12–36 Months***	
**Age (months), Average (SD)**	22.8 (7.3)
**Gender**	
Male	70 (52.2)
Female	64 (47.8)
Other	0 (0.0)
**Race/ethnicity**	
White	17 (12.7)
Black/African American	39 (29.1)
Hispanic/Latino	36 (26.9)
American Indian/Alaska Native	0 (0.0)
Asian/Pacific Islander	3 (2.2)
More than one race/ethnicity	36 (26.9)
Other	3 (2.2)

**Table 3:** Food group intake in past week by young children, n (%), shows need for dietary improvement (highlighted cells approximately meet dietary recommendations).

	Fruit	Vegetables	Juice	Sweetened	Snack Foods	Fast Food
				Beverages		
None	1	2	23	60	4	73
	(0.7%)	(1.5%)	(17.2%)	(44.8%)	(3.0%)	(54.5%)
1–2 times/week	11	18	33	35	84	61
	(8.2%)	(13.4%)	(24.6%)	(26.1%)	(62.7%)	(45.5%)
3–4 times/week	26	32	24	16	46	
	(19.4%)	(23.9%)	(17.9%)	(11.9%)	(34.3%)	
5–6 times/week	19	20	14	8		
	(14.2%)	(14.9%)	(10.4%)	(6.0%)		
Daily	77	62	40	15		
	(57.5%)	(46.3%)	(29.9%)	(11.2%)		

**Table 4:** Themes with sample quotes as reported in focus groups with caregivers of toddlers.

Theme	Quote
Lack of trust in child hunger or fullness cues	“Because they just keep eating. And they don’t know when to stop. And you can see her belly’s so full and you’re like, you need to stop eating. That’s not good. I don’t think they know”. “They see it there, they just keep eating. They see it there, they’ll just keep eating. Just keep eating and eating”. “Sometimes I feel like throughout today my granddaughter doesn’t get enough because either she doesn’t like it or she’s busy and she wants to play”.
Trust in child hunger or fullness cues	“She seems to know how much she wants to eat, because I can give her a little bag of snacks that I have in the bag and she will not eat it all. She knows automatically when she’s had enough”. “But if she doesn’t ask me to eat, I don’t give her anything. I don’t know, does that sound bad? But I figure, if she’s hungry, she’ll tell me. She’s old enough to”. “I think after a while, you feed them, you know how much they’re going to eat. You know what I’m trying to say? So if I know—if I have a piece of chicken and I’m cutting it up for her, I cut her just so much. And I know that’s what she’s going to eat”.
Using force and bribery to ensure children eat enough and a variety of foods	“And then we bribe her sometimes. We tell her, OK. You have two—not bribe her, but say, OK. You have to eat two more pieces of meat before you can have the mashed potatoes. And it usually works”. “So sometimes I go, and I’m like, [child name], you know, Mommy pays so much money for all this food. You have to eat. Eat so you be like Mommy. You’ll be big like Mommy”. “When they go to eat, they do not want to eat, then I take the tablet. And I tell you, until you eat you will not use the tablet”. “I could get them to do anything for a graham cracker and some fishies”.
RF-aligned strategies to encourage children to eat non-preferred foods	“I do try to schedule the meals and snacks. And the reason I do that is because I don’t want her to be hungry when I’m tied up doing something else” “So she’s sing all these little things. I’ll say ‘Try something new.’ We’ll do the little song, and then she’ll try it. And if she doesn’t like it, she doesn’t like it”. “What I heard about teaching children to eat more vegetables. Even if they don’t like it, you don’t force it. But you give them a small serving on their plate and they see you eat it. And then it takes a certain amount of time before they try it or what have you. I am desperately trying that”. “They won’t eat just plain broccoli, or they won’t eat just plain corn. They like their corn and their rice mixed in with stuff, like that. They’ll eat it like that”.

**Table 5:** Integrated results matrix depicting findings from surveys and focus groups with caregivers of toddlers.

Quantitative Results	Qualitative Results	Exemplar Quote
Child influences score positively associated with healthy intake	Lack of trust in child hunger or fullness cues	“And she says sometimes she doesn’t want to finish it all. But sometimes she prefers—I see she prefers rice than the vegetables. And I say, OK. You can leave the rice, but eat the vegetables”.
	Trust in child hunger or fullness cues	“He did not eat though, let’s say, for breakfast, so I give him fruits or something. I don’t force him”.
Environmental influences score positively associated with healthy intake	RF-aligned strategies to encourage children to eat non-preferred foods	“Because I put the snacks always in the bag—a little bag for her. Like grapes, strawberries, carrots. The little baby carrots. And oranges in little pieces. So she has many choices”. “She likes the pasta. And sometimes when I give her pasta, I just put the sauce—homemade sauce. Not only tomato. I mix meat with vegetables and then put it on the top of the pasta”.
Environmental influences score negatively associated with less healthy intake	Using force and bribery to ensure children eat enough and a variety of foods	“One thing that I have failed on with her is because she’s so sweet tooth addicted that I say—and I have done this and I have to find a way to stop—is that I say if you eat your healthy dinner, you can have a bowl of ice cream”.

## Data Availability

Data are available upon request.

## Supplementary Materials

The following supporting information can be downloaded at: https://www.mdpi.com/article/10.3390/nu16060863/s1, Table S1: Scoring of responsive feeding questions and conceptual groups; Figures S1 and S2. Focus group handouts.
