# Supplementary material for: Responsive Feeding Practices to Promote Healthy Diets: A Mixed Method Study among Low-Income Caregivers with Toddlers
Source: Nutrients. 2024 Mar 16;16(6):863. doi: 10.3390/nu16060863 (PMC10974279; doi:10.3390/nu16060863)
Supplement: Supplementary file 1 [file nutrients-16-00863-s001.zip › Supplemental Table.pdf]

<u>Supplemental Tables</u>

Responsive Feeding Conceptual Groups

Questions about responsive feeding (n=25) were placed into conceptual groups. Each question was scored 0-4, with greater scores indicating greater alignment with responsive feeding. Questions within each conceptual group were averaged, and thus, each conceptual group is also scored 0-4, with greater scores indicating alignment with responsive feeding.

Table S1. Scoring of responsive feeding questions and conceptual groups.

**Environmental Influences Score**	
**Question**	**Scale (Numerical Value)**
**Meal Environment (0-4)**	
My child ate with the rest of the family<br>I enjoy spending time with my child at mealtimes	Never in the past 7 days (0)<br>1 or 2 days (1)<br>3 or 4 days (2)<br>5 or 6 days (3)<br>Every day (4)
My child ate at the table or in a high chair	Never in the past 7 days (0)<br>1 or 2 days (1)<br>3 or 4 days (2)<br>5 or 6 days (3)<br>Every day (4)
My child ate while watching TV	Never in the past 7 days (4)<br>1 or 2 days (3)<br>3 or 4 days (2)<br>5 or 6 days (1)<br>Every day (0)
My child ate on the go, such as in a stroller or car seat, or on the bus	Never in the past 7 days (4)<br>1 or 2 days (3)<br>3 or 4 days (2)<br>5 or 6 days (1)<br>Every day (0)
I enjoy spending time with my child at mealtimes	Never in the past 7 days (0)<br>1 or 2 days (1)<br>3 or 4 days (2)<br>5 or 6 days (3)<br>Every day (4)
**Food offered and caregiver modeling (0-4)**	
Other people in my family make it hard for me to feed my child healthy	Disagree strongly (4)<br>Disagree (3)<br>Neutral (2)<br>Agree (1)<br>Agree Strongly (0)
The food we eat as a family provides enough nutrition for my child	Disagree strongly (0)<br>Disagree (1)<br>Neutral (2)

	Agree (3)<br>Agree Strongly (4)
**Parent Nutrition Beliefs (0-4)**	
Picky eaters need products like Pediasure, Enfagrow, or Nido to get enough nutrition	Disagree strongly (4)<br>Disagree (3)<br>Neutral (2)<br>Agree (1)<br>Agree Strongly (0)
Toddler formulas or powdered milks provide nutrition that children don't get from other food and drinks	Disagree strongly (4)<br>Disagree (3)<br>Neutral (2)<br>Agree (1)<br>Agree Strongly (0)
Pureed food that comes in pouches is a good way to teach toddlers to like the taste of fruits and vegetables	Disagree strongly (4)<br>Disagree (3)<br>Neutral (2)<br>Agree (1)<br>Agree Strongly (0)
Children won't eat the same food as the rest of the family. They need their own type of food	Disagree strongly (4)<br>Disagree (3)<br>Neutral (2)<br>Agree (1)<br>Agree Strongly (0)
100% juice is a good choice if a child won't eat fruit or vegetables	Disagree strongly (4)<br>Disagree (3)<br>Neutral (2)<br>Agree (1)<br>Agree Strongly (0)
Children should be served fruits and vegetables every day	Disagree strongly (0)<br>Disagree (1)<br>Neutral (2)<br>Agree (3)<br>Agree Strongly (4)
**Child Influences Score**	
**Child Self-Regulation of Intake (0-4)**	
[Picture of a meal shown] Is it like the amount of food you serve at a meal for your child?	More food than shown in the picture (0)<br>Same amount of food shown in the picture (4)<br>Less food than shown in the picture (2)
Who decides how much food your child eats?	Only me or another adult (0)<br>Mostly me or another adult (1)<br>Me or another adult and my child the same (2)<br>Mostly my child (3)<br>My child only (4)
Do you make your child finish all the food you serve?	Yes (0)<br>Sometimes (2)<br>No (4)
**Child Hunger and Satiety Cues (0-4)**	

My child tells me when he or she is hungry	All the time (4)<br>Most of the time (3)<br>Sometimes (2)<br>Rarely (1)<br>Never (0)
I worry that my child eats too much	Disagree strongly (4)<br>Disagree (3)<br>Neutral (2)<br>Agree (1)<br>Agree Strongly (0)
It is difficult to get my child to eat enough at meals	Disagree strongly (4)<br>Disagree (3)<br>Neutral (2)<br>Agree (1)<br>Agree Strongly (0)
**Food for Reward or Behavior (0-4)**	
It's OK to give children drinks with added sugar once in a while	Disagree strongly (4)<br>Disagree (3)<br>Neutral (2)<br>Agree (1)<br>Agree Strongly (0)
It would be mean to not give children sweet treats once in a while	Disagree strongly (4)<br>Disagree (3)<br>Neutral (2)<br>Agree (1)<br>Agree Strongly (0)
In the past 7 days, how many days did you give your child something to eat or drink because he or she was fussy?	Never in the past 7 days (4)<br>1 or 2 days (3)<br>3 or 4 days (2)<br>5 or 6 days (1)<br>Every day (0)
**Child Food Acceptance (0-4)**	
My child only eats a few foods	Disagree strongly (4)<br>Disagree (3)<br>Neutral (2)<br>Agree (1)<br>Agree Strongly (0)
My child will not taste a new food	Disagree strongly (4)<br>Disagree (3)<br>Neutral (2)<br>Agree (1)<br>Agree Strongly (0)
