# Supplementary figures and images for: Responsive Feeding Practices to Promote Healthy Diets: A Mixed Method Study among Low-Income Caregivers with Toddlers
Source: Nutrients. 2024 Mar 16;16(6):863. doi: 10.3390/nu16060863 (PMC10974279; doi:10.3390/nu16060863)

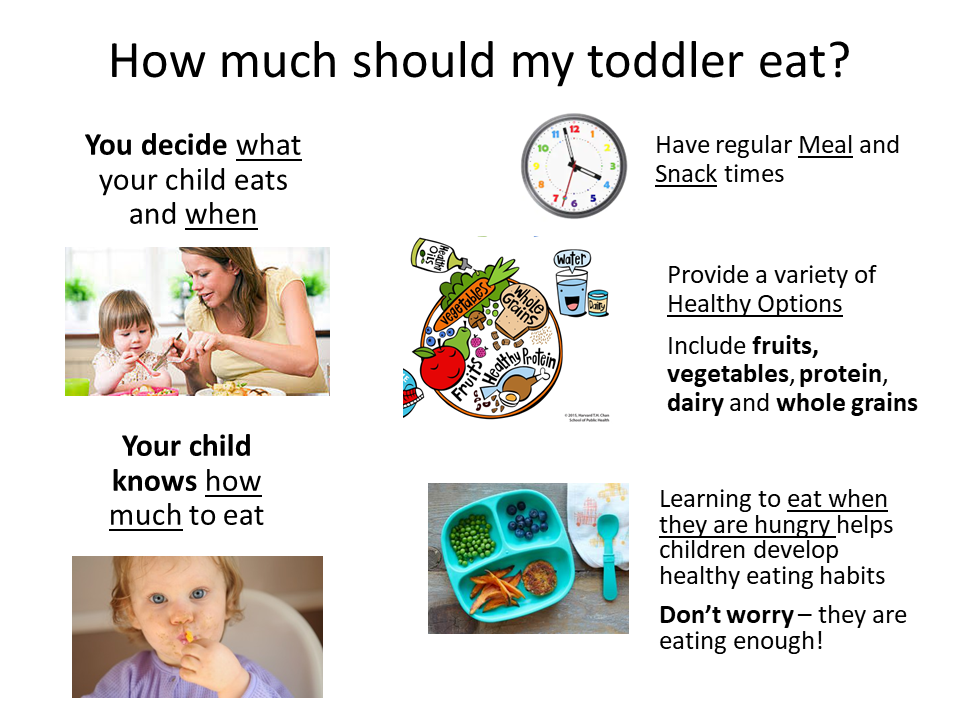

Supplement: Supplementary file 1 [file nutrients-16-00863-s001.zip › Supplemental Figure S1.png]

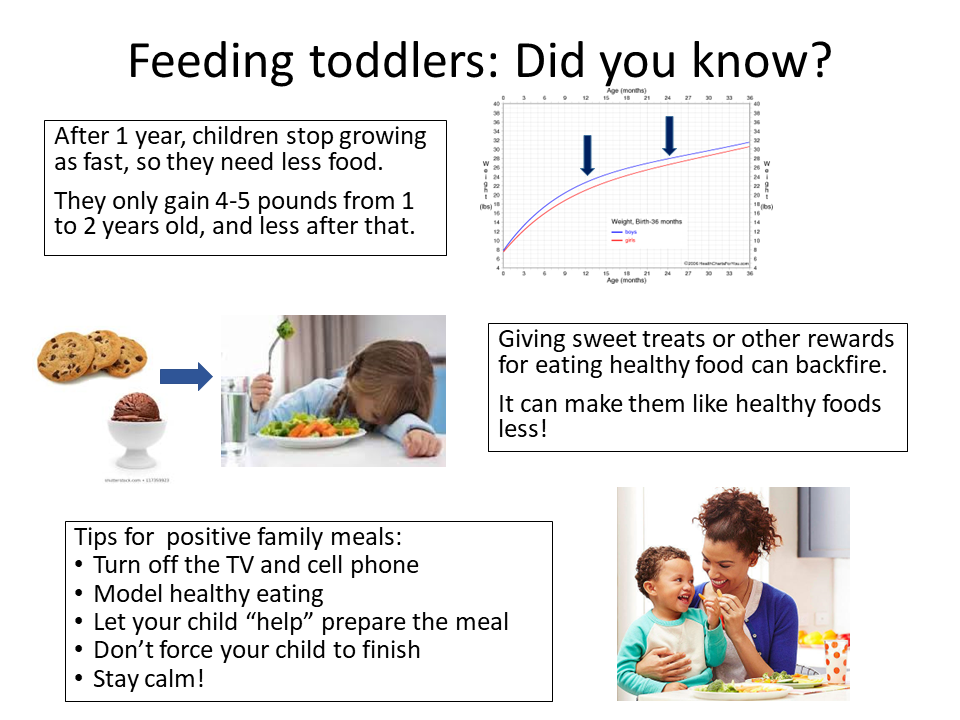

Supplement: Supplementary file 1 [file nutrients-16-00863-s001.zip › Supplemental Figure S2.png]
